# Comparison of HPV-16 and HPV-18 Genotyping and Cytological Testing as Triage Testing Within Human Papillomavirus–Based Screening in Mexico

**DOI:** 10.1001/jamanetworkopen.2019.15781

**Published:** 2019-11-20

**Authors:** Leticia Torres-Ibarra, Jack Cuzick, Attila T. Lorincz, Donna Spiegelman, Eduardo Lazcano-Ponce, Eduardo L. Franco, Anna Barbara Moscicki, Salaheddin M. Mahmud, Cosette M. Wheeler, Berenice Rivera-Paredez, Rubí Hernández-López, Leith León-Maldonado, Jorge Salmerón

**Affiliations:** 1 Center for Population Health Research, National Institute of Public Health, Cuernavaca, Mexico; 2 Wolfson Institute of Preventive Medicine Centre for Cancer Prevention, Queen Mary University, London, United Kingdom; 3 Center for Methods in Implementation and Prevention Science, Yale School of Public Health, New Haven, Connecticut; 4 Division of Cancer Epidemiology, McGill University, Montreal, Quebec, Canada; 5 David Geffen School of Medicine, University of California, Los Angeles; 6 Vaccine and Drug Evaluation Centre, Department of Community Health Sciences, University of Manitoba, Winnipeg, Manitoba, Canada; 7 Center for HPV Prevention, New Mexico Comprehensive Cancer Center, University of New Mexico, Albuquerque; 8 Research Center on Policies, Population, and Health, Faculty of Medicine, National Autonomous University of Mexico, Mexico City, Mexico; 9 Center for Population Health Research, Consejo Nacional de Ciencia y Tecnología, National Institute of Public Health, Cuernavaca, Mexico

## Abstract

**Importance:**

Triage tests enhance the efficiency cervical cancer screening based on human papillomavirus (HPV), but the best approach for maximizing programmatic effectiveness is still uncertain, particularly in a real-world scenario.

**Objective:**

To compare the clinical performance of 6 triage strategies based on liquid-based cytology (LBC) and HPV-16 and HPV-18 genotyping individually or in combination as sequential triage tests to detect cervical intraepithelial neoplasia (CIN) grade 2 or higher among women with high-risk HPV.

**Design, Setting, and Participants:**

This diagnostic study of routine cervical cancer screening was conducted at 100 primary health centers in Tlaxcala, Mexico. Women aged 30 to 64 years were recruited from August 1, 2013, to February 24, 2016, as part of the Forwarding Research for Improved Detection and Access for Cervical Cancer Screening and Triage study. Six triage scenarios for referral to colposcopy were examined: (1) LBC testing that found atypical squamous cells of undetermined significance (ASC-US) or worse, (2) positive results in HPV-16 genotyping, (3) positive results in HPV-18 genotyping, (4) positive results in HPV-16/HPV-18 genotyping, (5) positive results in HPV-16 genotyping or, if genotyping results were negative, reflex LBC testing that found ASC-US or worse, and (6) positive results in HPV-16/HPV-18 genotyping or, if genotyping results were negative, reflex LBC testing that found ASC-US or worse. Data were analyzed from October 2017 to August 2018.

**Exposures:**

Liquid-based cytological testing with simultaneous HPV-16 and HPV-18 genotyping. Women whose HPV genotyping results were positive for HPV-16 or HPV-18 or whose LBC results found ASC-US or worse and a random set of negative and normal results were referred to colposcopy with histologic analysis used for disease confirmation.

**Main Outcomes and Measures:**

Clinical performance of each test strategy for detection of CIN grade 2 or higher. Secondary outcomes included resource utilization of each triage scenario, measured by the number of tests performed, the referral rate for colposcopy, and the numbers of colposcopies per CIN grade 2 or higher detected.

**Results:**

A total of 36 212 women (median [interquartile range] age, 40 [35-47] years) were screened, and 4051 women (11.2%) had high-risk HPV. Of these women, 1109 (24.6%) were found to have HPV-16, HPV-18, or ASC-US or worse. Further histologic testing detected CIN grade 2 or higher in 110 of 788 women (14.0%) who underwent follow-up colposcopy. Sensitivity and specificity for 3 main triage strategies were 42.9% and 74.0% for LBC; 58.3% and 54.4% for HPV-16/HPV-18 genotyping; and 86.6% and 34.0% for HPV-16/HPV-18 genotyping with reflex LBC. The referral rate to colposcopy was 29% for HPV-16/HPV-18 with reflex LBC, which was 2-fold higher than the referral rate of 12% for LBC.

**Conclusions and Relevance:**

Triage of women with high-risk HPV with HPV-16/HPV-18 genotyping with reflex LBC was significantly associated with improvement in detection of CIN grade 2 or higher compared with LBC alone. The benefit of disease prevented may outweigh the cost of increasing requirements for colposcopy services in settings with limited adherence to follow-up after a positive screening result.

## Key Points

**Question:**

What combination of human papillomavirus 16 (HPV-16) and HPV-18 genotyping with liquid-based cytological (LBC) testing is associated with the best performance for triaging women with HPV to detect cervical intraepithelial neoplasia ?

**Findings:**

This diagnostic study included 36 212 participants in the Forwarding Research for Improved Detection and Access for Cervical Cancer Screening and Triage study and found that a combined triage strategy of HPV-16/HPV-18 genotyping with reflex LBC was associated with an increase in the relative sensitivity to detect cervical intraepithelial neoplasia grade 2 or higher compared with LBC testing alone.

**Meaning:**

The combination of HPV-16/HPV-18 genotyping with reflex LBC may offer a reliable strategy for discriminating women at greater risk of cervical cancer, avoiding unnecessary anxiety and diagnostic procedures in primary cervical cancer screening among women with high-risk HPV.

## Teaser

This diagnostic study compares the efficiency and effectiveness of 6 triage testing scenarios for screening women with high-risk human papillomavirus (HPV) for referral for colposcopy.

## Introduction

It has been estimated that 311 365 deaths worldwide were due to cervical cancer in 2018, with 90% occurring in developing countries.^1^ While the lack of screening is likely the primary reason for these deaths, the availability of promising new screening technologies underscores the need for evaluation and rapid scale-up implementation of the best options at the population level.^2^

Although screening represents only one piece of the whole process, its clinical performance and associated operational attributes, such as health care infrastructure or quality assurance requirements, have a critical role in effectiveness of cervical cancer control.^3,4^ Human papillomavirus (HPV) testing as a primary screening tool for cervical cancer has been shown to have favorable attributes, including higher throughput, greater sensitivity, and better reproducibility than standard cytological examination.^5,6,7^ For countries lacking successfully integrated cytology screening, such as low- to middle-income countries (LMICs), the World Health Organization^8^ recommends HPV testing as the preferred choice for cervical cancer screening.

A primary concern of testing for high-risk HPV, defined as an HPV infection with any of the 12 types of HPV that are known to cause cancer (also known as *oncogenic HPV types*), is its low positive predictive value, since only a small fraction of women with high-risk HPV will have a persistent infection that is likely to progress to invasive cancer.^9^ Therefore, referring all women with high-risk HPV to colposcopy would likely lead to overtreatment and incur high health care costs. A question that remains is the nature of the best combination of triage tests for cervical cancer screening using primary HPV testing.^10,11^ Recently, HPV-16/HPV-18 genotyping or cytological examination have been recommended to triage for immediate colposcopy in some national screening guidelines from high-income countries, offering greater sensitivity than cytological examination alone and better specificity than referring all women with HPV for colposcopy.^12,13,14^ Nevertheless, the suitability of these strategies in the routine health care practice in other resource-constrained settings has not been explored, to our knowledge. Country-based type-specific HPV prevalence and genotype distribution in cervical precancerous lesions,^15^ as well as the health care infrastructure relevant to LMICs, could influence the performance of these triage strategies.^4^

The Mexican Ministry of Health commissioned the National Institute of Public Health of Mexico (INSP) to assess the performance and program components of alternatives for triaging women with HPV prior to integrating them into the national cervical screening program. In 2013, INSP launched the Forwarding Research for Improved Detection and Access for Cervical Cancer Screening and Triage (FRIDA) study, a demonstration project of HPV-based screening, that included the introduction of several tests for triaging to colposcopy with the goal of strengthening the current guidelines, which were launched in 2009. In this study, we aimed to compare the clinical test performance of the current triage tests available in Mexico, liquid-based cytology (LBC) testing and HPV-16 or HPV-18 genotyping, individually or in combination as sequential tests to detect cervical intraepithelial neoplasia (CIN) grade 2 or higher, among women with high-risk HPV participating in a routine cervical cancer screening program.

## Methods

The FRIDA study was approved by the institutional review boards of INSP, Tlaxcala State Ministry of Health, and the Mexican Federal Commission for the Protection Against Sanitary Risk. All participants provided verbal informed consent because the study involved no more than minimal risk and the same procedures of routine cervical cancer screening were used. This report adhered to the Standards for Reporting of Diagnostic Accuracy (STARD) reporting guideline.^16^

### Study Population

The detailed methods of the FRIDA study have been described elsewhere.^17^ Briefly, the FRIDA study was conducted as part of routine cervical cancer screening within Tlaxcala Health Services, which serve a population insured by Seguro Popular, a public health insurance program that offers coverage to all residents of Mexico not protected by any other social security institution. The target population for the FRIDA study was women aged 30 to 64 years who visited 100 primary health centers in 29 municipalities, representing half of Tlaxcala, Mexico’s population of 126 335 women in this age group.

The study population included all women who attended for routine cervical cancer screening at one of these clinics between August 1, 2013, and February 24, 2016. Women with a hysterectomy or who were pregnant at the time of screening were excluded. Verbal informed consent was obtained by health care personnel after explaining the nature of the study; those who agreed were administered a survey to collect demographic, gynecological, and obstetric data.

### Outcome Measures

The primary outcome was the cross-sectional performance of 6 triage strategies for detection of CIN grade 2 or higher in women with high-risk HPV. Outcomes were examined according to 6 triage scenarios for referring to colposcopy: (1) LBC examination that found atypical squamous cells of undetermined significance (ASC-US) or worse, (2) positive results in HPV-16 genotyping, (3) positive results in HPV-18 genotyping, (4) positive results in HPV-16/HPV-18 genotyping, (5) positive results in HPV-16 genotyping or, if genotyping results were negative, reflex LBC testing that found ASC-US or worse, and (6) positive results in HPV-16/HPV-18 genotyping or, if genotyping results were negative, reflex LBC testing that found ASC-US or worse. Relative sensitivity and specificity of each triage strategy were estimated compared with LBC. The resource utilization for each triage strategy was a secondary outcome.

### Study Procedures

Each participant underwent a pelvic exam to collect 2 cervical samples using 2 separate cervical brushes (Rovers). One brush was preserved in PreservCyt vial (Hologic) for HPV DNA and other molecular testing. The other brush was placed in a SurePath vial (Becton, Dickinson and Company) for LBC testing.

#### HPV Testing

Vials for HPV DNA testing were transported to the INSP HPV laboratory in Cuernavaca, Mexico, and stored at 2°C to 8°C until testing could take place. Samples were tested for high-risk HPV using the cobas 4800 HPV test (Roche Molecular Diagnostics), a qualitative in vitro assay that identifies a pooled result for 12 high-risk HPV types, including HPV-31, HPV-33, HPV-35, HPV-39, HPV-45, HPV-51, HPV-52, HPV-56, HPV-58, HPV-59, HPV-66, and HPV-68, and individual results for HPV-16 and HPV-18.

#### LBC

The vials used for LBC testing were stored at 2°C to 8°C upon arrival at the central cytology laboratory of Tlaxcala Health Services in Tlaxcala, Mexico. Samples were processed using the PrepStain System (TriPath Imaging) according to the manufacturer’s instructions. Slides were stained with the Papanicolaou method and interpreted according to the Bethesda 2001 criteria.^18^ The threshold used to define abnormal cytologic results was ASC-US or worse.

Liquid-based cytology slides were reviewed by 2 independent cytotechnologists who were blinded to their counterpart’s result. If both cytotechnologists reported an abnormality, the worst result was established as the final diagnosis, otherwise a cytopathologist determined the final diagnosis. The cytopathologist also evaluated 5% of the slides with normal findings and all slides with ASC-US findings or worse, in compliance with the Mexican Cervical Cancer Screening program. Both the cytotechnologists and the cytopathologist were aware that all slides under evaluation were positive results for high-risk HPV but were blinded to HPV-16 and HPV-18 status.

#### Diagnosis Confirmation

Women whose test results were positive in HPV-16 or HPV-18 genotyping or for whom LBC examination found ASC-US or worse were offered colposcopy, as well as randomly chosen women (29%) whose test results were negative for HPV-16/HPV-18 genotyping and whose LBC findings were normal, to correct for partial verification bias.

All colposcopies were performed by 4 trained colposcopists in 2 colposcopy clinics, Tlaxcala’s General Hospital and Women’s Hospital. The colposcopic findings were reported according to the 2011 International Federation of Cervical Pathology and Colposcopy guidelines.^19^ Colposcopists were aware of the women’s triage test results and medical history. In addition, colposcopists collected a minimum of 4 biopsies (1 per quadrant of the squamocolumnar junction) and an endocervical sample in all women regardless of the colposcopic findings.

These biopsy specimens were processed at the central pathology laboratory in Tlaxcala General Hospital. Pathological diagnosis served as the criterion standard for the diagnosis of cervical precancer and cancer. Histological evaluation was performed by a panel of pathologists who reported the diagnosis according to Mexico’s Cervical Cancer Screening Program’s criteria.^20^ If the diagnoses of the first 2 pathologists agreed, that was the final diagnosis; otherwise, biopsies were read in a blinded fashion by a third pathologist for end point adjudication. Clinical data collection was completed in October 2017.

### Statistical Analysis

Descriptive statistics were used to summarize baseline characteristics. We evaluated the diagnostic performance of the triage strategies among participants with high-risk HPV in the FRIDA study using the baseline screening results.

Two disease end points were assessed: (1) CIN grade 2 or higher, defined as women with CIN grade 2, CIN grade 3, or cancer confirmed via histologic analysis and (2) CIN grade 3 or higher, defined as CIN grade 3 or cancer confirmed via histologic analysis. For each triage scenario, we calculated sensitivity, specificity, positive predictive value, and negative predictive value, with 95% CIs using a generalized estimating equation (GEE) approach with an exchangeable working correlation structure to account for the within-woman correlation in triage results.^21^ Relative sensitivities were also estimated from the GEE method with a log link function, using LBC alone as the comparator, which is the triage test used in the current screening program in Mexico.

By design, partial verification bias occurred in our study, as only a small fraction of women with high-risk HPV who had a negative HPV-16 and HPV-18 genotyping results and normal LBC examination results were verified by histologic analysis, leading to missing data on disease outcome and biased estimates of diagnostic performance.^22,23,24^ To obtain verification bias adjusted (VBA) estimates of sensitivity and specificity as if all women with high-risk HPV had received the criterion standard, a weighted GEEs method was used.^21^ This method incorporates a weight representing the inverse probability of undergoing the criterion standard test, that is, the inverse of the sampling fraction of receiving the criterion standard test within women with high-risk HPV, either to allow VBA in women whose triage tests were negative and normal or to account for nonadherence among women with positive or abnormal triage test results referred to colposcopy.

The McNemar test for discordant pairs was used to test the differences in sensitivity and specificity between paired triage results within CIN grade 2 or higher and CIN less than grade 2 groups. A McNemar *P* value of more than .05 was assumed to indicate that the sensitivity or specificity of the index algorithm was not significantly different from the comparator.^25^

To assess resource utilization of each triage scenario, we measured the number of tests performed, the referral rate for colposcopy, and the numbers of colposcopies per CIN grade 2 or higher detected. The numbers of colposcopies per CIN grade 2 or higher detected is an indicator of the diagnostic efficiency provided by each triage test, calculated as number of the histologically-confirmed CIN grade 2 or higher divided by the number of colposcopies performed.^24^ All analyses were 2-tailed, and a *P* value of less than .05 was considered significant. Statistical calculations were performed using Stata software version 14.1 (StataCorp). Data were analyzed from October 2017 to August 2018.

## Results

A total of 36 212 women were screened in the FRIDA study. The median (interquartile range [IQR]) age of participants was 40 (35-47) years (Table 1), with significantly higher participation at younger ages (*P* for trend < .001). Most women were married or cohabiting (31 995 women [88.4%]), with age at sexual debut at least 18 years in 23 026 women (63.6%). The median (IQR) number of lifetime sexual partners was 1 (1-2) partner, with 25 408 women (70.2%) reporting having had only 1 lifetime partner. Most women had had at least 1 pregnancy (35 313 women [97.5%]), and nearly half had had 3 or 4 pregnancies (17 381 women [48.0%]). Only 101 women (0.3%) reported having received at least 1 HPV vaccine dose, and 14 977 women (41.4%) reported having undergone a Papanicolaou test within the previous 3 to 5 years (Table 1).

**Table 1.** Characteristics of the Study Population and Prevalence of High-Risk HPV Infection

Characteristics	Women, No. (%) (N = 36 212)	Prevalence of High-Risk HPV, % (95% CI) (n = 4051)^a^
Age, y		
Median (IQR)	40 (35-47)	NA
30-34	8299 (22.9)	14.1 (13.3-14.8)
35-39	8289 (22.9)	11.4 (10.7-12.1)
40-44	7210 (19.9)	9.6 (8.9-10.3)
45-49	5202 (14.4)	8.9 (8.1-9.7)
50-54	3556 (9.8)	10.4 (9.4-11.4)
55-59	2388 (6.6)	11.3 (10.0-12.5)
60-64	1268 (3.5)	11.4 (9.7-13.2)
*P* value for trend	<.001	
Marital status		
Single	2204 (6.1)	17.7 (16.2-19.3)
Married or cohabitating	31 995 (88.4)	10.3 (10.0-10.7)
Divorced or separated	1102 (3.0)	18.9 (16.6-21.2)
Widowed	612 (1.7)	17.3 (14.3-20.3)
Missing	299 (0.8)	14.7 (10.7-18.7)
Age of sexual debut, y		
Median (IQR)	18 (17-21)	
<18	13 125 (36.2)	12.1 (11.6-12.7)
≥18	23 026 (63.6)	10.7 (10.3-11.1)
Missing	61 (0.2)	8.2 (1.3-15.1)
Lifetime sexual partners, No.		
Median (IQR)	1 (1-2)	
1	25 408 (70.2)	9.0 (8.7-9.4)
2	6992 (19.3)	15.0 (14.1-15.8)
3-5	3322 (9.2)	19.4 (18.0-20.7)
≥6	339 (0.9)	21.5 (17.2-25.9)
Missing	151 (0.4)	0.6 (0.6-2.0)
Pregnancies, No.		
0	899 (2.5)	15.7 (13.3-18.1)
1-2	8857 (24.5)	12.3 (11.6-13.0)
3-4	17 381 (48.0)	10.4 (10.0-10.9)
5-9	8513 (23.5)	11.0 (10.4-11.7)
≥10	532 (1.5)	12.8 (9.9-15.6)
Missing	30 (0.1)	6.7 (2.3-15.6)
Current IUD use		
No	25 349 (65.0)	11.2 (10.8-11.6)
Yes	11 806 (32.6)	11.7 (11.1-12.2)
Missing	857 (2.4)	5.3 (3.8-6.7)
History of hormonal contraception use		
Ever	7387 (20.4)	12.5 (11.7-13.2)
Never	28 524 (78.8)	10.9 (10.6-11.3)
Missing	301 (0.8)	4.7 (2.3-7.0)
Smoking history		
Currently	766 (2.1)	18.8 (16.0-21.6)
Former	405 (1.1)	16.8 (13.2-20.4)
Never	34 346 (94.8)	11.1 (10.8-11.4)
Missing	695 (1.9)	4.5 (2.9-6.0)
Self-reported HIV status		
Positive	129 (0.4)	27.1 (10.5-34.8)
Papanicolaou test history		
Never	7400 (20.4)	13.0 (12.2-13.8)
In past 3-5 y	14 977 (41.4)	10.1 (9.6-10.5)
In past 18 mo	13 293 (36.7)	11.5 (10.9-12.0)
Missing	542 (1.5)	10.5 (7.9-13.1)
HPV test history		
Never	24 842 (68.6)	11.4 (11.0-11.8)
In past 5 y	10 464 (28.9)	10.6 (10.1-11.2)
Missing	906 (2.5)	11.2 (9.1-13.2)

Abbreviations: HPV, human papillomavirus; IQR, interquartile range; IUD, intrauterine device; NA, not applicable.

^a^ Calculated according to row total for each category of each variable.

The STARD flow diagram is presented in the eFigure in the Supplement. A total of 4051 women (11.2%) had high-risk HPV. All women with high-risk HPV had valid test results for HPV-18 and all but 3 women had valid test results for HPV-16. The prevalence of HPV-16 was 13.3%, and the prevalence for HPV-18 was 6.0%. The overall combined prevalence of HPV-16 or HPV-18 was 18.2%. Among 3949 women (97.5%) with valid LBC results, 472 women (11.9%) had LBC findings that indicated the presence of ASC-US or worse. Missing LBC data included 10 inadequate specimens and 92 women without available results.

Of 3962 women with high-risk HPV and complete triage results, 1109 women (28%) had at least 1 positive or abnormal triage result, and 102 women (2.5%) had test results that were positive for HPV-16 or HPV-18 and ASC-US or worse cytologic abnormality. Among 1109 women with positive results for at least 1 of the triage tests, 788 women (71.1%) underwent colposcopy and had complete histological test results. The remaining 2853 women (70.4%) had negative results on HPV-16 and HPV-18 genotyping tests and normal findings on LBC tests, from which 366 of 818 women randomly chosen for VBA underwent colposcopy and had histological results. The characteristics of women in the control group for VBA and the rest of the women with negative or normal results on all triage tests are presented in eTable 2 in the Supplement. In total, 110 CIN grade 2 or higher were found in women with a positive test result for any triage test, including 35 CIN grade 2, 69 CIN grade 3, and 6 invasive cervical cancers. Among women with negative or normal test results for all triage tests, 17 CIN grade 2 or higher were found, including 7 CIN grade 2, 9 CIN grade 3, and 1 invasive cervical cancer. Table 2 presents the distribution of histological outcomes according to triage strategy in women who underwent colposcopy. Overall, a greater proportion of women with CIN grade 2 or higher had positive or abnormal results on triage tests compared with women whose triage test results were negative and normal, except for the HPV-18 triage strategy. Although CIN grade 2 or higher cases were detected in women with negative results for all triage tests, a lower proportion of CIN grade 2 or higher was observed in women with negative results to the sequential triage scenarios (ie, HPV-16 genotyping with reflex LBC and HPV-16/HPV-18 genotyping with reflex LBC) compared with the disease detected in negative results from triage scenarios based on individual testing (ie, LBC, HPV-16 genotyping, HPV-18 genotyping, or HPV-16/HPV-18 genotyping alone). There were 72 women (9%) with CIN grade 2 or higher among those who had normal LBC findings, compared with 17 women (4.6%) with CIN grade 2 or higher among women with negative results in HPV-16/HPV-18 tests and normal LBC results.

**Table 2.** Distribution of Histological Outcomes Stratified by Triage Test Results Among Women Who Underwent Colposcopy

Triage Results	Total Biopsies, No.^a^	Biopsy Finding, No. (%)				
		Normal	CIN Grade 1	CIN Grade 2	CIN Grade 3	Invasive Cervical Cancer
Liquid-based cytology						
ASC-US or worse	320	95 (29.7)	171 (53.4)	17 (5.3)	35 (10.9)	2 (0.6)
ASC-US	66	16 (24.2)	43 (65.2)	2 (3.0)	5 (7.6)	0
Low-grade SIL	182	61 (33.5)	100 (54.9)	11 (6.0)	10 (5.5)	0
High-grade SIL	62	16 (25.8)	27 (43.5)	2 (3.2)	15 (24.2)	2 (3.2)
Squamous cell carcinoma	4	0	0	0	4 (100)	0
Atypical glandular cells	6	2 (333)	1 (16.7)	2 (33.3)	1 (16.7)	0
Normal	828	337 (40.7)	419 (50.6)	25 (3.0)	42 (5.1)	5 (0.6)
HPV-16 genotyping						
Positive	396	139 (35.1)	192 (48.5)	21 (5.3)	39 (9.8)	5 (1.3)
Negative	757	294 (38.8)	402 (53.1)	20 (2.6)	39 (5.2)	2 (0.3)
HPV-18 genotyping						
Positive	182	77 (42.3)	92 (50.5)	3 (1.6)	10 (5.5)	0
Negative	974	357 (36.7)	503 (51.6)	39 (4.0)	68 (7.0)	7 (0.7)
HPV-16/HPV-18 genotyping						
Positive	543	204 (37.6)	265 (0.48.8)	23 (4.2)	46 (8.5)	5 (0.9)
Negative	613	230 (37.5)	330 (53.8)	19 (3.1)	32 (5.2)	2 (0.3)
HPV-16 genotyping with reflex LBC						
Positive and reflex LBC findings ASC-US or worse^b^	665	223 (33.5)	339 (51.0)	33 (5.0)	64 (9.6)	6 (0.9)
Reflex LBC findings normal	485	209 (43.1)	253 (52.2)	8 (1.6)	14 (2.9)	1 (0.2)
HPV-16/HPV-18 genotyping with reflex LBC						
Positive and reflex LBC findings ASC-US or worse^b^	788	283 (35.9)	395 (50.1)	35 (4.4)	69 (8.8)	6 (0.8)
LBC findings normal^c^	366	151 (41.3)	198 (54.1)	7 (1.9)	9 (2.5)	1 (0.3)

Abbreviations: ASC-US, atypical squamous cells of undetermined significance; CIN, cervical intraepithelial neoplasia; HPV, human papillomavirus; LBC, liquid-based cytology; SIL, squamous intraepithelial lesion.

^a^ Only histologically confirmed biopsies were included. Women were offered colposcopy if they had positive or abnormal results to any triage test, but only 71% of women with any positive result underwent follow-up colposcopy for disease verification.

^b^ Includes women whose HPV genotyping results were positive and women whose HPV genotyping results were negative and therefore underwent reflex LBC testing.

^c^ Includes women who had negative results in HPV-16/HPV-18 genotyping and normal findings in LBC and who belonged to the group selected to correct for verification bias and accepted undergoing to disease verification (12%).

### Clinical Performance of Different Triage Scenarios to Detect CIN Grade 2 or Higher and Grade 3 or Higher

To allow comparisons between different pairs of triage tests, a cross-tabulation is presented as eTable 1 in the Supplement showing results by CIN status. Table 3 shows the performance of different triage strategies in detection of CIN grade 2 or higher and CIN grade 3 or higher. Liquid-based cytological examination had a sensitivity of 42.9% (95% CI, 34.1%-52.0%), which was a lower sensitivity value compared with HPV-16 genotyping alone (51.6%; 95% CI, 42.5%-60.6%) and HPV-16/HPV-18 genotyping (58.3%; 95% CI, 49.1%-67.0%). There was a greater relative sensitivity ratio of HPV-16/HPV-18 combined genotyping (1.4; 95% CI, 1.01-1.82; *P* = .04) compared with LBC for detecting CIN grade 2 or higher. Conversely, LBC had higher specificity for CIN grade 2 or higher (74.0%; 95% CI, 71.2%-76.6%) than HPV-16/HPV-18 genotyping (54.4%, 95% CI, 51.3%-57.5%). The highest sensitivities for detection of CIN grade 2 or higher were found using the sequential algorithms of HPV-16 genotyping with reflex LBC (81.7%; 95% CI, 73.9%-88.1%) and HPV-16/HPV-18 genotyping with reflex LBC (86.6%; 95% CI, 79.4%-92-0%), although a comparison between these two sequential tests showed that HPV-16/HPV-18 genotyping with reflex LBC had statistically significantly higher sensitivity (McNemar *P* = .03). Lower specificities were observed in HPV-16 genotyping with reflex LBC (45.1%; 95% CI, 42.0%-48.2%) and HPV-16/HPV-18 genotyping with reflex LBC (34.0%; 95% CI, 31.0%-37.0%) compared with LBC as single test for triage. The Figure presents a plot of sensitivity vs false-positive results (calculated as 1 − specificity). In general, the combination of HPV genotyping and LBC examination as sequential tests avoided the loss in sensitivity but had a proportional increase in false-positives. Similar data were obtained with CIN grade 3 or higher as the end point (Table 3). The positive predictive values of CIN grade 2 or higher among women whose test results were positive or abnormal in any of triage tests were between 7.1% (95% CI, 3.9%-11.9%) for HPV-18 genotyping alone to 16.9% (95% CI, 12.9%-21.4%) for LBC (Table 3).

**Table 3.** Diagnostic Performance of Triage Strategies to Identify CIN Grade 2 or Higher or Grade 3 or Higher Among Women With High-Risk HPV

Triage Scenario	Result, % (95% CI)						Relative Sensitivity (95% CI)^b^
	Sensitivity		Specificity		PPV	NPV	
	Crude	Adjusted^a^	Crude	Adjusted^a^			
**Detection of CIN Grade ≥2**							
LBC	42.9 (34.1-52.0)	26.7 (19.7-36.1)	74.0 (71.2-76.6)	89.7 (88.3-91.1)	16.9 (12.9-21.4)	91.3 (89.2-93.1)	1 [Reference]
HPV-16 genotyping	51.6 (42.5-60.6)	32.2 (24.3-42.7)	67.8 (64.8-70.6)	87.3 (85.8-88.9)	16.4 (12.9-20.4)	91.9 (89.8-93.8)	1.2 (0.89-1.64)
HPV-18 genotyping	10.2 (5.6-16.9)	6.4 (3.6-11.2)	83.6 (81.2-85.8)	93.5 (92.5-94.6)	7.1 (3.9-11.9)	88.3 (86.1-90.2)	0.24 (0.13-0.43)
HPV-16/HPV-18 genotyping	58.3 (49.1-67.0)	36.2 (27.1-46.4)	54.4 (51.3-57.5)	82.1 (80.2-84.0)	13.6 (10.9-16.8)	91.4 (88.8-93.5)	1.4 (1.01-1.83)
HPV-16 genotyping with reflex LBC	81.7 (73.9-88.1)	50.9 (40.0-64.8)	45.1 (42.0-48.2)	78.4 (76.3-80.7)	15.5 (12.8-18.5)	95.3 (93.0-97.0)	1.9 (1.59-2.29)
HPV-16/HPV-18 genotyping with reflex LBC	86.6 (79.4-92.0)	53.8 (42.5-68.1)	34.0 (31.1-37.0)	74.1 (71.6-76.6)	14.0 (11.6-16.6)	95.4 (92.7-97.3)	2.0 (1.7-2.4)
**Detection of CIN Grade ≥3**							
LBC findings ASC-US or worse	44.0 (33.2-55.3)	28.7 (20.0-41.3)	73.4 (70.6-76.0)	89.4 (88.0-90.7)	11.6 (8.3-15.6)	94.3 (92.5-95.8)	1 [Reference]
HPV-16 genotyping	51.8 (40.7-62.7)	33.7 (24.0-47.3)	67.0 (64.1-69.9)	86.8 (82.3-88.4)	11.1 (8.1-14.6)	94.6 (92.7-96.1)	1.17 (0.81-1.70)
HPV-18 genotyping	11.8 (5.79-20.6)	7.6 (4.0-14.5)	83.9 (81.6-86.1)	93.6 (92.6-94.6)	5.5 (2.7-9.9)	92.3 (90.4-93.9)	0.27 (0.14-0.52)
HPV-16/HPV-18 genotyping	60.0 (48.8-70.5)	39.1 (28.3-53.8)	54.1 (51.0-57.1)	81.7 (79.8-83.7)	9.4 (7.1-12.2)	94.5 (92.3-96.1)	1.4 (0.96-1.93)
HPV-16 genotyping with reflex LBC	82.4 (72.6-89.8)	53.6 (40.2-71.5)	44.1 (41.1-47.2)	77.8 (75.6-80.0)	10.5 (8.3-13-1)	96.9 (95.0-98.3)	1.86 (1.50-2.32)
HPV-16/HPV-18 genotyping with reflex LBC	88.2 (79.4-94.2)	57.4 (43.4-76.1)	33.3 (30.5-36.2)	73.5 (71.1-76.0)	9.5 (7.6-11.8)	97.3 (95.0-98.7)	2.0 (1.6-2.5)

Abbreviations: CIN, cervical intraepithelial neoplasia; HPV, human papillomavirus; LBC, liquid-based cytology; NPV, negative predictive value; PPV, positive predictive value.

^a^ Estimates are corrected for partial verification bias within women with HPV who underwent triage tests.

^b^ Relative sensitivity compared with LBC testing using atypical squamous cells of undetermined significance or worse findings as the positivity threshold.

**Figure.**
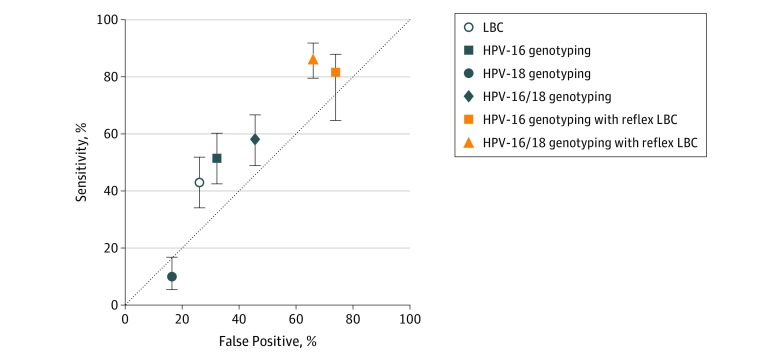
Sensitivity vs False-Positives for Triage Strategies to Detect Cervical Intraepithelial Neoplasia Grade 2 or Higher Cytology findings atypical squamous cells of undetermined significance or worse were the threshold set for abnormal liquid-based cytology (LBC). Error bars indicate 95% CIs; and HPV, human papillomavirus.

Of all triage alternatives, the sequential triage tests were associated with the highest proportion of colposcopy referrals, including 939 referrals of 4048 women (23.2%) with the HPV-16 genotyping with reflex LBC triage scenario and 1109 referrals of 3962 women (28.0%) with the HPV-16/HPV-18 with reflex LBC triage scenario, whereas LBC alone resulted in 472 referrals of 3949 women (12.0%) (Table 4). The strategy of HPV-16/HPV-18 genotyping with reflex LBC yielded the highest detection of CIN grade 2 or higher, with 110 cases detected, which was more than 2-fold that of referrals from LBC alone (54 cases). Furthermore, only 1 invasive cancer were not detected using the reflex triage approach. However, the strategy of HPV-16/HPV-18 genotyping and reflex LBC required 7.2 colposcopies to detect 1 CIN grade 2 or higher, the highest number of colposcopies, compared with 5.9 required by LBC alone.

**Table 4.** Diagnostic Efficiency for Colposcopy Referrals and Disease Detection by Triage Strategy

Triage Scenario	Tests Performed, No^a^	Colposcopy Referrals, No./Women Tested, No. (%)	Women With Complete Diagnosis Confirmation, No. (%)^b^	No.	
				CIN Grade ≥2 Detected	Colposcopies to Detect 1 CIN Grade **≥**2
LBC^c^	3949	472/3949 (12.0)	320 (67.8)	54	5.9
HPV-16 genotyping^d^	4048	540/4048 (13.3)	396 (73.3)	65	6.1
HPV-18 genotyping	4051	244/4051 (6.0)	182 (74.6)	13	14.0
HPV-16/HPV-18 genotyping	4051	739/4051 (18.2)	543 (73.5)	74	7.3
HPV-16 genotyping with reflex LBC	7466	939/4048 (23.2)	665 (70.8)	103	6.5
HPV-16/HPV-18 genotyping with reflex LBC	7274	1109/3962 (28.0)	788 (71.1)	110	7.2

Abbreviations: CIN, cervical intraepithelial neoplasia; HPV, human papillomavirus; LBC, liquid-based cytology.

^a^ The number of tests performed in all women with high-risk HPV (n = 4051) corresponds to the number of samples tested from which valid results were obtained. In the triage strategies combining HPV genotyping with reflex LBC, cervical samples of women whose results were negative for the first triage test were referred on to the second test (LBC) using atypical squamous cells of undetermined significance or worse as the positivity threshold for LBC.

^b^ Includes women who underwent colposcopy and had successful histological evaluations.

^c^ Although by protocol all women with high-risk HPV should had been tested by LBC, valid LBC results were available only in 97.7% of women with high-risk HPV. There were 102 women with missing LBC results: 10 women had an inadequate specimen and 92 women did not have available results.

^d^ There were 3 samples with invalid results for HPV-16 genotyping but with valid results for HPV-18 genotyping, therefore they met the criteria for colposcopy referral (ie, positive results for HPV-16 or HPV-18).

## Discussion

One of the most pressing issues in cervical cancer screening is finding adequate triage methods for patients with high-risk HPV. Thus, we assessed the clinical performance of LBC, HPV-16 genotyping, and HPV-18 genotyping for detection CIN grade 2 or higher using 6 different algorithms. The use of sequential testing based on HPV-16/HPV-18 genotyping followed by reflex LBC offered the highest sensitivity but the lowest specificity for detection of CIN grade 2 or higher compared with LBC alone, findings that corroborate those of previous cross-sectional screening trials.^26,27,28,29^ A sequential triage algorithm for screening women with high-risk HPV with HPV-16/HPV-18 genotyping as the first test may be a practical alternative for organizing HPV-based screening programs. Most validated high-risk HPV tests yield HPV-16/HPV-18 results as part of the same testing process and HPV-16/HPV-18 genotype testing is more reproducible than cytological analysis. Moreover, it has been demonstrated that presence of HPV-16 is a major factor for risk of CIN grade 3 or higher.^30,31^ A 2016 cohort study^31^ found that HPV-16 and HPV-18 are associated with the greatest 3-year risk of CIN grade 3 or higher.

It is important to highlight that although a different biopsy protocol was applied in the Addressing the Need for Advanced HPV Diagnostics (ATHENA) HPV study^26^ (ie, a random biopsy vs systematic collection of 4 biopsies plus endocervical sampling performed in the FRIDA study), our performance estimates for triage based on HPV genotyping were very similar. According to a study by Mayrand et al,^32^ sensitivity of HPV testing is less likely to be affected by identification of more incipient lesions through a multibiopsy approach. In contrast, we observed a more striking difference for the LBC triage sensitivity, with 42.9% in our study vs 52.6% obtained in the ATHENA HPV study.^26^ Assuming that colposcopic findings are what influence a colposcopist to target cervical areas for biopsy, visible and mostly larger lesions would likely be associated with the higher sensitivity estimates for LBC found in earlier studies. Additionally, although we conducted a prestudy training for interpreting LBC slides, subjectivity plays an important role in the performance of the tests, as noted by a subanalysis of the ATHENA HPV study^26^ by Castle et al.^33^ Problems inherent with misclassification of cytologic diagnosis, either by clinician factors, such as the variability of technical training and experience of our cytotechnologists, or by the cytological scoring itself, may also contribute to these differences.

Our analysis found that decreasing the screening start age to offer HPV-based screening rather than LBC from 35 years to 30 years may be associated with an increase in the detection of precancerous lesions, as this age group contributed to one-third of the total CIN grade 2 or higher observed. Although this finding suggests the use of a more sensitive screening method, a key concern is the need to be less aggressive in CIN detection and management considering the potential for adverse reproductive outcomes associated with treatment of precancerous lesions in young women.^34^

An important implication of our findings for the application of these triage approaches in other settings is in the utilization of diagnostic resources. The HPV-16/HPV-18 genotyping with reflex LBC strategy was associated with an increase in the detection of CIN grade 2 or higher but also increased 2-fold the number of colposcopy referrals compared to LBC alone. Although the economic costs for follow-up, colposcopy, and treatment of false positives may seem sufficient reason to accept the failure to detect some CIN grade 2 or higher, especially if a significant proportion of lesions will spontaneously regress, these may be offset by the economic and social cost savings of preventing the occurrence of some cancers.

Since women in resource-poor settings have a low probability of getting in contact with prevention services,^35^ we believe that treating a woman at high risk of cervical cancer will produce a better outcome for women’s overall health and for public health in general in the long term. Moreover, screening algorithms with low sensitivity require frequent repetitions of screening, which pose logistical barriers in LMICs that have barriers to adherence to follow-up recommendations as well as inequity in access to services.^36,37^

A more accurate detection of cancer progression through novel specific biomarkers based on DNA methylation or cell cycle dynamics might be influential in decisions to refer to or defer treatment.^12,38^ Indeed, as vaccinated cohorts enter into screening, such biomarkers will be imperative to adjust clinical practices in accordance with the change in HPV patterns and the increasing number of less relevant lesions.^39^ For now, while HPV vaccination programs leave adult women unprotected, strategies based on HPV-16 and HPV-18 combined genotyping or LBC may offer an acceptable performance for triaging women with high-risk HPV.

This study is the first large study evaluating triage alternatives in real-world LMIC conditions, to our knowledge. Although training was conducted prior to the beginning of our study, the study procedures were performed within a routine screening program, providing information about the triage performance in real-world environments. Our findings may thus be generalizable to populations with similar characteristics. The design of the FRIDA study permitted an efficient and unbiased comparison of triage tests and their algorithms. One of the main strengths was that our study included a systematic collection of biopsies, reducing the potential bias of differential disease misclassification associated with the colposcopic impression. It is worth noting that disease ascertainment was by pathology panel review, which was led by an experienced pathologist and attained a consensus diagnosis in all cases.

### Limitations

Our study had some important limitations. The FRIDA study was not powered to detect differential test performance characteristics by subgroups. We could not ascertain if some CIN grade 2 or CIN grade 3 that were detected at baseline may have been transient lesions, resulting in outcome misclassification.^40^ This measurement error could be higher in the group of women whose results were negative in HPV-16/HPV-18 genotyping and normal in LBC who were used for VBA, considering that CIN grade 2 lesions caused by high-risk HPV types other than HPV-16 or HPV-18 are more likely to regress.^30,32,41,42^ A 2016 study^31^ indicated that the 3-year risk of CIN grade 3 or higher ranged from 1.2% to 4.0% for patients with high-risk HPV who did not have HPV-16 or HPV-18 and had normal cytological findings. This can lead to an underestimation of the true sensitivity and specificity of the analysis if using VBA. Although VBA estimates were reported to ensure comparability with other screening trials, our results should be interpreted with caution to prevent dissemination of misleading data about the potential benefit of a particular triage strategy.

## Conclusions

This diagnostic study found that HPV-16/HPV-18 genotyping with reflex LBC for triaging women with high-risk HPV is an appropriate method to achieve the goal of preventing cervical cancer in settings where access to appropriate follow-up is challenging, assuming that the costs and burden of overtreatment are offset by the individual and social benefits of preventing cervical cancer. Perceptions and preferences of women and health care professionals, as well as the resources required, are key factors that should be addressed to ensure effective screening programs.

## Notes

Supplement. **eTable1. **Number of Discordant Pairs and McNemar Test for Discordant Pairs Between Different Triage Strategies Among Cases of Cervical Intraepithelial Neoplasia **eTable 2.** Characteristics of Women With Negative or Normal Triage Test Results Women Stratified by Receipt of Disease Verification by Histopathological Examination to Adjust for Verification Bias **eFigure.** STARD Diagram
