# Supplementary material for: Comparison of HPV-16 and HPV-18 Genotyping and Cytological Testing as Triage Testing Within Human Papillomavirus–Based Screening in Mexico
Source: JAMA Netw Open. 2019 Nov 20;2(11):e1915781. doi: 10.1001/jamanetworkopen.2019.15781 (PMC6902808; doi:10.1001/jamanetworkopen.2019.15781)
Supplement: Supplement. — eTable1. Number of Discordant Pairs and McNemar Test for Discordant Pairs Between Different Triage Strategies Among Cases of Cervical Intraepithelial Neoplasia eTable 2. Characteristics of Women With Negative or Normal Triage Test Results Women Stratified by Receipt of Disease Verification by Histopathological Examination to Adjust for Verification Bias eFigure. STARD Diagram [file jamanetwopen-2-e1915781-s001.pdf]

Supplementary Online Content

Torres-Ibarra L, Cuzick J, Lorincz AT, et al. Comparison of HPV-16 and HPV-18 genotyping and cytological testing as triage testing within human papillomavirus–based screening in Mexico. *JAMA Netw Open*. 2019;2(11):e1915781. doi:10.1001/jamanetworkopen.2019.15781

eTable 1. Number of Discordant Pairs and McNemar Test for Discordant Pairs Between Different Triage Strategies Among Cases of Cervical Intraepithelial Neoplasia
eTable 2. Characteristics of Women With Negative or Normal Triage Test Results Women Stratified by Receipt of Disease Verification by Histopathological Examination to Adjust for Verification Bias
eFigure. STARD Diagram

This supplementary material has been provided by the authors to give readers additional information about their work.

© 2019 Torres-Ibarra L et al. *JAMA Network Open.*

eTable 1. Number of Discordant Pairs and McNemar Test for Discordant Pairs Between Different Triage Strategies Among Cases of Cervical Intraepithelial Neoplasia

			Test1/Test2 result			Crude				
**Test 1**	**Test 2**	**Total (n)**	**- / -**	**+/-**	**-/+**	**+/+**	**Sens (test1)**	**Sens (test2)**	**p-value McNemar[a]**	**McNemar mid-p test****
**CIN2+**										
HPV16	LBC	125	23	48	38	16	51.6 (42.5-60.6)	42.9 (34.1-52.0)	.28	-
HPV18	LBC	126	61	11	52	2	10.2 (5.6-16.9)	42.9 (34.1-52.0)	<.001	-
HPV16/18	LBC	126	17	55	36	18	58.3 (49.1-67.0)	42.9 (34.1-52.0)	.046	-
HPV16+ → reflex LBC (ASCUS+) if HPV16-	LBC	125	23	48	0	54	81.7 (73.9-88.1)	42.9 (34.1-52.0)	<.001	<.001
HPV16/18+ → reflex LBC if HPV 16/18 -	LBC	126	17	55	0	54	86.6 (79.4-92.0)	42.9 (34.1-52.0)	<.001	<.001
HPV16	HPV16/18	125	53	0	8	64	51.6 (42.5-60.6)	58.3 (49.1-67.0)	0.008	.004
HPV16+ → reflex LBC (ASCUS+) if HPV16-	HPV16/18	125	17	36	6	66	81.7 (73.9-88.1)	58.3 (49.1-67.0)	<.001	<.001
HPV16/18+ → reflex LBC if HPV 16/18 -	HPV16/18	126	17	36	0	73	86.6 (79.4-92.0)	58.3 (49.1-67.0)	<.001	<.001
HPV16/18+ → reflex LBC if HPV 16/18 -	HPV16+ → reflex LBC (ASCUS+) if HPV16-	125	17	6	0	102	86.6 (79.4-92.0)	81.7 (73.9-88.1)	.03	.02
**<CIN2+**							**Spe (test1)**	**Spe (test2)**		
HPV16	LBC	1020	462	292	231	35	67.8 (64.8-70.6)	74.0 (71.2-76.6)	.008	-
HPV18	LBC	1022	615	141	240	26	83.6 (81.2-85.8)	74.0 (71.2-76.6)	<.001	-
HPV16/18	LBC	1022	349	407	209	57	54.4 (51.3-57.5)	74.0 (71.2-76.6)	<.001	-
HPV16+ → reflex LBC (ASCUS+) if HPV16-	LBC	1020	462	292	0	266	45.1 (42.0-48.2)	74.0 (71.2-76.6)	<.001	<.001

© 2019 Torres-Ibarra L et al. *JAMA Network Open.*

			Test1/Test2 result				Crude			
**Test 1**	**Test 2**	**Total (n)**	**- / -**	**+/-**	**-/+**	**+/+**	**Sens (test1)**	**Sens (test2)**	**p-value McNemar a**	**McNemar mid-p test****
HPV16/18+ → reflex LBC if HPV 16/18 -	LBC	1022	349	407	0	266	34.0 (31.1-37.0)	74.0 (71.2-76.6)	<.001	<.001
HPV16	HPV16/18	1027	560	0	136	331	67.8 (64.8-70.6)	54.4 (51.3-57.5)	<.001	<.001
HPV16+ → reflex LBC (ASCUS+) if HPV16-	HPV16/18	1020	349	209	113	349	45.1 (42.0-48.2)	54.4 (51.3-57.5)	<.001	-
HPV16/18+ → reflex LBC if HPV 16/18 -	HPV16/18	1022	349	209	0	464	34.0 (31.1-37.0)	54.4 (51.3-57.5)	<.001	<.001
HPV16/18+ → reflex LBC if HPV 16/18 -	HPV16+ → reflex LBC (ASCUS+) if HPV16-	1020	349	113	0	558	34.0 (31.1-37.0)	45.1 (42.0-48.2)	<.001	<.001

Abbreviation: hrHPV, high-risk human papilloma virus; LBC, Liquid-based cytology; CIN2+, cervical intraepithelial neoplasia grade 2 or worst. [a]p-value McNemar asymptotic when the frequency of discordant pairs is greater than 10, otherwise McNemar exact is reported. [b]McNemar mid-p test. Note: The number of histological outcomes differs by each combination of triage tests according to the availability of valid complete results of them. For instance, there were 102 women hrHPV+ with missing LBC results: 10 had an inadequate specimen and 92 with results not available. There were 3 samples with invalid results for HPV16 genotyping but with valid results for HPV18, therefore they met the criteria to colposcopy referral (positive infection for HPV16 and/or HPV18).

© 2019 Torres-Ibarra L et al. *JAMA Network Open.*

eTable 2. Characteristics of Women With Negative or Normal Triage Test Results Women Stratified by Receipt of Disease Verification by Histopathological Examination to Adjust for Verification Bias

Characteristics		Total triage negative (N=2853)[a]	Control group Verified n=366[b] (12.8%)	No verified n=2,487 (87.2%)	p-value[c]
		% (No.)	% (95% CI)	% (95% CI)	
Age (years)					
	Median (IQR)	39 (34-47)	39 (34-46)	39 (34-48)	.39
	30-34	27.6 (788)	29.2 (24.6-33.9)	27.4 (25.6-29.1)	.46
	35-39	23.0 (657)	24.6 (20.2-29.0)	22.8 (21.1-24.4)	.45
	40-44	17.0 (484)	16.9 (13.1-20.8)	17.0 (15.5-18.4)	>.99
	45-49	11.6 (330)	10.9 (7.7-14.1)	11.7 (10.4-12.9)	.68
	50-54	9.9 (282)	8.7 (5.8-11.6)	10.1 (8.9-11.2)	.43
	55-59	7.4 (210)	6.6 (4.0-9.1)	7.5 (6.4-8.5)	.53
	60-64	3.6 (102)	3.0 (1.3-4.8)	3.7 (2.9-4.4)	.53
Age of sexual debut (years)					
	Median (IQR)	18 (16-20)	18 (16-20)	18 (16-20)	.59
Number of Lifetime sexual partners					
	Median (IQR)	1 (1-2)	1 (1-2)	1 (1-2)	.12
	1 partner	57.8 (1649)	53.5 (48.4-58.7)	58.4 (56.5-60.4)	.078
	2 partners	25.5 (726)	29.0 (24.3-33.6)	24.9 (23.2-26.6)	.098
	3-5 partners	15.1 (432)	15.6 (11.9-19.3)	15.1 (13.7-16.5)	.80
	6 or more partners	1.6 (46)	1.9 (0.5-3.3)	1.6 (1.1-2.1)	.62
Number of sexual partners in the last 12 months					
	0 partners	20.4 (583)	22.5 (18.1-26.9)	20.1 (18.6-21.7)	.31
	1 partner	78.5 (2239)	77.2 (72.7-81.6)	78.6 (77.0-80.3)	.54
	2 or more	1.0 (27)	0.29 (-0.3-0.9)	1.0 (.6-1.4)	.18
	Missing	0.1 (4)	0	0.2 (0.0-0.3)	.46
Number of pregnancies					
	Median (IQR)	3 (2-5)	3 (2-5)	3 (2-5)	.025
	None	3.4 (96)	3.8 (1.9-5.8)	3.3 (2.6-4.0)	.60
	1-2	26.6 (759)	30.9 (26.1-35.6)	26.0 (24.2-27.7)	.048
	3-4	44.4 (1266)	44.3 (39.2-49.3)	44.4 (42.4-46.3)	.96
	5-9	24.1 (687)	19.7 (15.6-23.7)	24.7 (23.0-26.4)	.035
	10 or more	1.5 (44)	1.4 (0.2-2.5)	1.6 (1.1-2.0)	.77
Number of vaginal deliveries					
	None	25.8 (736)	28.1 (23.5-32.7)	25.4 (23.7-27.2)	.27
	1-2	28.9 (798)	29.8 (25.1-34.5)	27.7 (25.9-29.5)	.41
	3-5	36.7 (1047)	33.6 (28.8-38.4)	37.1 (35.2-39.0)	.19
	6 or more	9.5 (271)	8.5 (5.6-11.3)	9.6 (8.5-10.8)	.47
History of Hormonal Contraception					
	No	77.0 (2196)	80.9 (76.8-84.9)	76.4 (74.7-78.1)	.057
	Yes	22.7 (647)	19.1 (15.1-23.1)	23.2 (21.5-24.8)	.082
	Missing	0.3 (10)	0	0.4 (0.1-0.6)	.22
Condom use					
	Never	78.6 (2242)	78.7 (74.5-82.9)	78.6 (76.9-80.2)	.96
	Almost always	10.9 (312)	10.6 (7.5-13.8)	11.0 (9.7-12.2)	.85

© 2019 Torres-Ibarra L et al. *JAMA Network Open.*

	Always	7.6 (216)	7.1 (4.5-9.7)	7.6 (6.6-8.7)	.72
	Missing	2.9 (83)	3.5 (1.6-5.4)	2.8 (2.2-3.5)	.43
Tobacco use					
	Never	94.4 (2693)	96.2 (94.2-98.1)	94.1 (93.2-95.0)	.11
	Former	1.3 (37)	0.8 (-0.1-1.7)	1.4 (0.9-1.8)	.39
	Current	3.6 (103)	2.5 (0.9-4.0)	3.8 (3.0-4.5)	.21
	Missing	0.7 (20)	0.5 (-0.2-1.3)	0.7 (0.4-1.0)	.70
History of Previous HPV screening					
	Never	69.7 (1989)	68.8 (64.1-73.6)	69.8 (68.0-71.6)	.70
	Yes	28.0 (798)	29.8 (25.1-34.5)	27.7 (25.9-29.5)	.41
	Missing	2.3 (66)	1.4 (0.2-2.5)	2.4 (1.8-3.1)	.20
History of Previous Pap screening					
	Never	23.0 (655)	25.1 (20.7-29.6)	22.6 (21.0-24.3)	.29
	Yes	75.7 (2159)	72.4 (67.8-77.0)	76.1 (74.5-77.8)	.12
	Missing	1.4 (39)	2.5 (0.9-4.0)	1.2 (0.8-1.6)	.054
History of previous cervical cancer screening					
	Never	21.4 (609)	22.4 (18.1-26.7)	21.2 (19.6-22.8)	.60
	Yes	77.4 (2207)	75.1 (70.7-79.6)	77.7 (76.0-79.3)	.28
	Missing	1.3 (37)	2.5 (0.9-4.0)	1.1 (0.7-1.5)	.035

[a] All HPV+ women with double negative triage tests results (negative to HPV16/18 tests and normal in cytology). [b] Subsample of women negative to LBC and HPV16/18 genotyping tests who receive disease verification used for correction of verification bias. [c] p-value two sample test of proportions for categorical variables or Two-sample Wilcoxon rank-sum (Mann-Whitney) test for continuous data.

© 2019 Torres-Ibarra L et al. *JAMA Network Open.*

eFigure. STARD Diagram

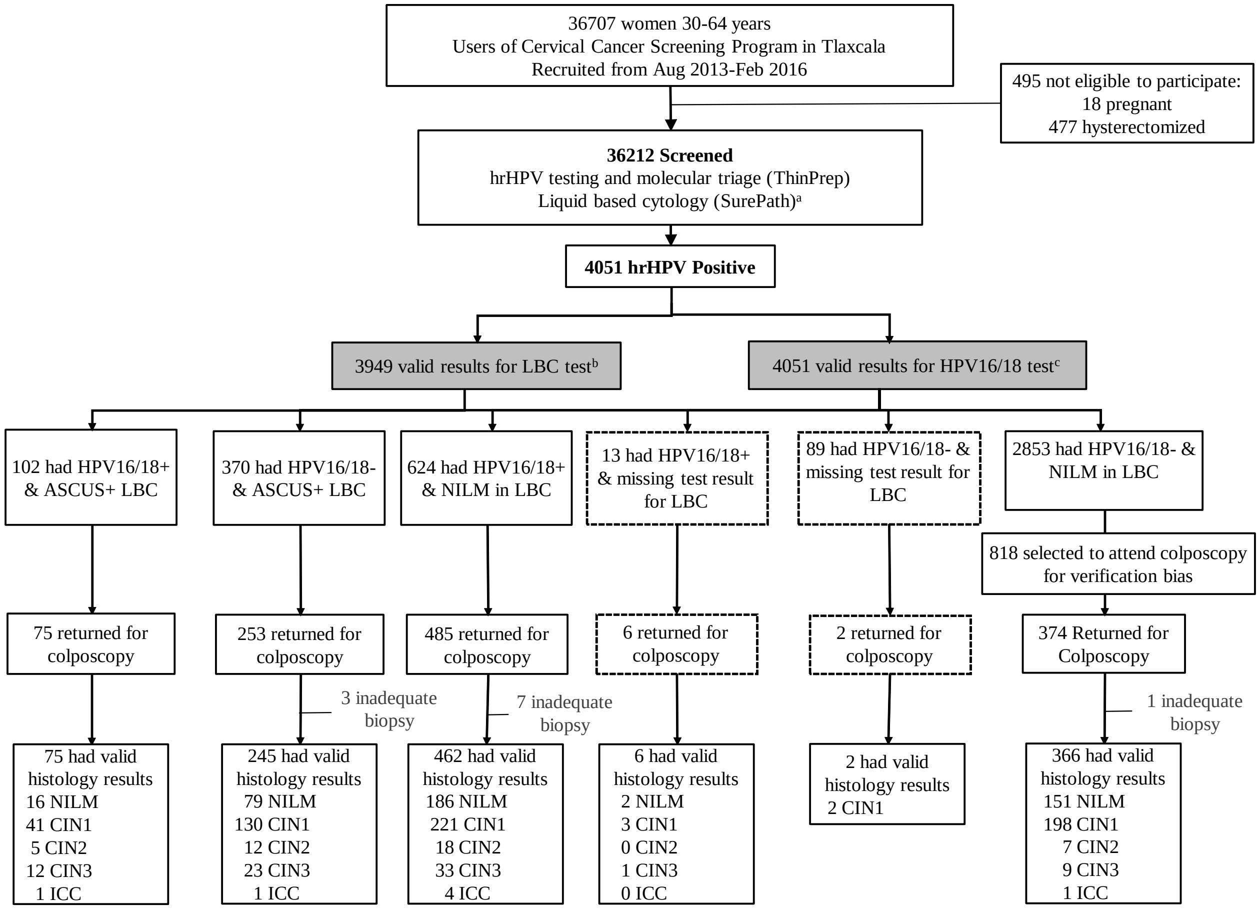

This flowchart represents the study design used to evaluate HPV16/18 genotyping and liquid-based cytology as triage tests using the baseline results of the first screening round of the FRIDA study trial.

Abbreviation: hrHPV, high-risk human papilloma virus; LBC, Liquid-based cytology; NILM, negative for intraepithelial lesion or malignancy, ASCUS+, Atypical squamous cells of undetermined significance or worse; CIN, cervical intraepithelial neoplasia; ICC, invasive cervical cancer.

[a]Sample placed in SurePath vial was only used to perform LBC in hrHPV positive women. [b]Although by protocol all hrHPV positive women should had been tested by LBC, valid LBC results were available only in 97.7% of hrHPV positive women. There were 102 hrHPV+ women with missing LBC results: 10 had an inadequate specimen and 92 did not have available results. [c]There were 3 samples with invalid results for HPV16 genotyping but with valid results for HPV18, therefore they met the criteria for colposcopy referral (positive infection for HPV16 and/or HPV18). Positive for HPV16/18 is indicated by HPV16/18+. Negatives for HPV16/18 are indicated by HPV16/18-. The boxes with dotted lines depict the women who were part of the HPV16/18 performance evaluation but who were excluded from the triage approach based on LBC.

© 2019 Torres-Ibarra L et al. *JAMA Network Open.*
